# Estimation of genetic diversity in Gute sheep: pedigree and microsatellite analyses of an ancient Swedish breed

**DOI:** 10.1186/s41065-017-0026-4

**Published:** 2017-01-30

**Authors:** Christina M. Rochus, Anna M. Johansson

**Affiliations:** 1 Department of Animal Breeding and Genetics, Faculty of Veterinary Medicine and Animal Science, Swedish University of Agricultural Sciences, Box 7023, Uppsala, SE-75007 Sweden; 2 UFR Génétique, Élevage et Reproduction; Sciences de la Vie et Santé, AgroParisTech, Université Paris-Saclay, Paris, France; 3 Génétique Physiologie Systèmes d’Elevage (GenPhySE), Université de Toulouse, INRA, INPT, ENVT, Castanet-Tolosan, France

## Abstract

**Background:**

Breeds with small population size are in danger of an increased inbreeding rate and loss of genetic diversity, which puts them at risk for extinction. In Sweden there are a number of local breeds, native breeds which have adapted to specific areas in Sweden, for which efforts are being made to keep them pure and healthy over time. One example of such a breed is the Swedish Gute sheep. The objective of this study was to estimate inbreeding and genetic diversity of Swedish Gute sheep.

**Results:**

Three datasets were analysed: pedigree information of the whole population, pedigree information for 100 animals of the population, and microsatellite genotypes for 94 of the 100 animals. The average inbreeding coefficient for lambs born during a six year time period (2007–2012) did not increase during that time period. The inbreeding calculated from the entire pedigree (0.038) and for a sample of the population (0.018) was very low. Sheep were more heterozygous at the microsatellite markers than expected (average multilocus heterozygosity and Ritland inbreeding estimates 1.01845 and -0.03931) and five of seven microsatellite markers were not in Hardy Weinberg equilibrium due to heterozygosity excess. The total effective population size estimated from the pedigree information was 155.4 and the average harmonic mean effective population size estimated from microsatellites was 88.3. Pedigree and microsatellite genotype estimations of inbreeding were consistent with a breeding program with the purpose of reducing inbreeding.

**Conclusion:**

Our results showed that current breeding programs of the Swedish Gute sheep are consistent with efforts of keeping this breed viable and these breeding programs are an example for other small local breeds in conserving breeds for the future.

**Electronic supplementary material:**

The online version of this article (doi:10.1186/s41065-017-0026-4) contains supplementary material, which is available to authorized users.

## Background

Gute sheep are an ancient breed from the Swedish island and province of Gotland, belonging to the North European short-tailed sheep group. Both Gute sheep and in general North European short-tailed sheep are characterized in part by their hardiness as well as coat colour and pattern variation [1]. Many of the North European short-tailed sheep breeds are decreasing in population size [1, 2] and have low heterogeneity [3]. However these breeds are unique and their conservation is relevant because they add to overall species diversity [3, 4]. The Gute sheep population experienced a severe bottleneck when polled sheep started to become more popular on Gotland and the horned sheep became rare. Beginning around 1920 the remaining horned sheep on Gotland were gathered by a few individuals interested in preserving the horned sheep. These sheep are the origins of the existing Gute sheep population today (personal communication with Gute sheep breed organizations in Sweden). Nevertheless, Gute sheep, unlike some other North European short-tailed sheep breeds, are not endangered at the moment, although the population is believed to be decreasing in size [2]. Thanks to conservation efforts, there were an estimated 5200 animals in Sweden in 2012 [2]. The first association working with the conservation of the Gute sheep, Föreningen Gutefåret, was formed in 1977, and an additional association, GutefårAkademin, was formed in 2007. Gute sheep breeding is focused on preserving the breed and not on improving production traits.

Gute sheep appear in the scientific literature, having been studied for both coat colour and population structure and genetic diversity. Classical genetic studies from the 1970s took advantage of the coat colour variation in Gute sheep, also then referred to as Goth sheep in English, to study coat colour inheritance in sheep [5]. More recently, there have been population genetic studies which have included Gute sheep [3, 6], but these studies only had a small number of individuals; 20 and 12 respectively. There have been no studies of Gute sheep genetic diversity using pedigree information.

The objectives of this study were to estimate inbreeding and genetic diversity of Swedish Gute sheep using pedigree data and microsatellite genotypes to identify; if the population is at potential risk due to high or increasing inbreeding and decreased genetic diversity; and if strategies need to be implemented to conserve the Swedish Gute sheep in the future.

## Methods

### Data

Pedigree information for all registered Gute sheep in Sweden born from 1960 to 2014 was provided by Elitlamm, the recording program for sheep in Sweden. For the early records only a small proportion of the population were registered in ElitLamm, but in recent years a very large proportion of the Gute sheep population in Sweden has been registered in ElitLamm because animals must be registered in order to be entitled to financial support for the keeping of endangered breeds. Pedigree information included animal identification numbers, year of birth and parent identification numbers. The animal identification numbers were anonymous.

Blood samples and phenotypic records (tail length and information about horns) were collected from different parts of Sweden by a trained technician in collaboration with the association “Föreningen Gutefåret”, one of the two Gute sheep breed organizations in Sweden. Ethical permission to collect blood samples from sheep and use for genetic studies was approved before the study (Dnr C102/13). Samples were stored in the -80 °C freezer of the SLU biobank. DNA was extracted from blood samples for 100 Gute sheep from a total of 13 flocks using the DNeasy mini kit for the QIAsymphony robot (Qiagen®, Hilden, Germany). Sheep were genotyped for a total of eight genetic markers; AME, a SNP marker in the amelogenin gene for sex determination; and seven microsatellite markers (INRA005, INRA023, INRA063, INRA172, MAF214, MAF65 and McM527). These markers are part of the ISAG kit for parentage testing. Genotyping was done at the certified lab at the Department of Animal Breeding and Genetics, SLU. The pedigree information for the 100 animals included flock identification, date of birth and official animal identification numbers for each animal, and their ancestors (which did not correspond with the anonymous animal identification numbers of the pedigree of the whole population).

### Analyses

Pedigree information of the whole population was used to estimate population parameters. Generation intervals, effective number of founders, effective number of ancestors, effective number of founder genomes and marginal contributions of ancestors were estimated with the software Pedig [7]. Generation interval for sires of sires, dams of sires, sires of dams and dams of dams were calculated separately. The average was calculated from these four values and used as the overall generation interval. The coefficient of inbreeding by birth year [8], pedigree completeness indices, number of complete generation equivalents and average coancestry within birth cohorts were estimated with software, EVA [9]. Effective population size was calculated as one divided by double the annual rate of inbreeding multiplied by the generation interval [10].

The pedigree information for each of the 100 sheep with blood samples available were combined into one pedigree. This pedigree was used for calculating average coefficient of inbreeding and pedigree completeness with the software EVA [9] for comparison with results for these parameters from the whole pedigree.

Diversity within populations (1-Q_INTRA_) and between populations (1-Q_INTER_), inbreeding in individuals compared with the population inbreeding (F_IS_), and Hardy Weinberg tests for heterozygosity deficiency and excess in microsatellite markers were estimated with the program Genepop v.4.3 [11]. Inbreeding and relatedness among individuals [12] were estimated using the microsatellite genotypes using software Coancestry v.1.0.1.5 [13]. Multilocus heterozygosity was calculated as the proportion of typed loci at which an individual was heterozygous divided by the population heterozygosity at those same loci [14]. Past changes in effective population size (average harmonic mean effective population size) were estimated using microsatellite genotypes in the software VarEff v.1.2 [15] with an assumed mutation rate of 0.00013 [16] and generation interval 3 years. Population structure was estimated with the microsatellite genotypes using principal components analysis (PCA) using R package adegenet [17].

## Results

**Fig. 1:**
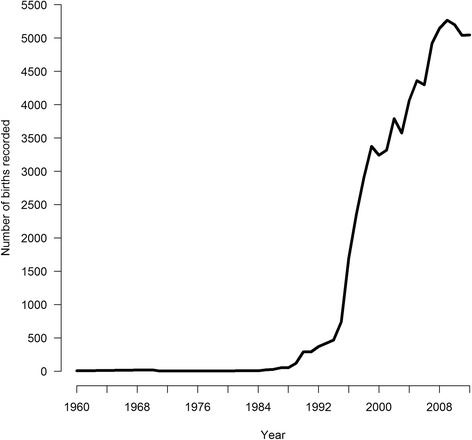
Total number of Gute sheep recorded in Sweden by birth year

**Fig. 2:**
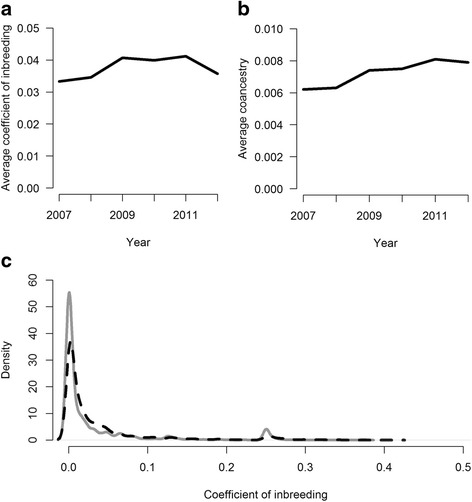
**a** Average inbreeding coefficient, **b** average coancestry for Gute sheep by birth year from 2007-2012 and **c** distribution of inbreeding coefficients of animals born in 2007 (solid grey line) and 2012 (dashed black line)

A total of 70 474 Gute sheep births were recorded in our dataset since 1960, with 30 616 recorded births during the last six years (2007 to 2012) (Fig. 1). Animals born from 2007 to 2012 had a pedigree completion index for three generations greater than 0.8. The average generation interval for this cohort was 3.6 years with generation intervals for sires being longer than for dams (Table 1), the total effective population size was 155.4 (Table 1) and the average inbreeding and average coancestry were low (0.038 and 0.007) (Fig. 2a and b). The distributions of inbreeding coefficients of animals born in 2007 and 2012 are in Fig. 2c. Measures of diversity and changes in population structure including effective number of founders, effective number of ancestors and effective number of founder genomes are reported in Table 1. From 1994 to 2003, the average inbreeding coefficient increased every year (from 0.0003 in 1994 to 0.037 in 2003), however, from 2003 to 2012 the average inbreeding coefficient remained relatively constant.

**Table 1:** Generation interval (L) and effective population size (N_e_) for Gute sheep in Sweden between 2007 and 2012 calculated using pedigree

	Males	Females
Generation interval (L) in years	3.7	3.5
Effective population size (N_e_)	152.8	157.7
Number of founders	575	715
Effective number of founders	204.6	225.7
Effective number of founder genomes	45.5	52.8
Effective number of ancestors	109.6	122.1

Number of founders, effective number of founders, effective number of founder genomes and effective number of ancestors calculated using pedigree in all recorded Swedish Gute sheep

**Fig. 3:**
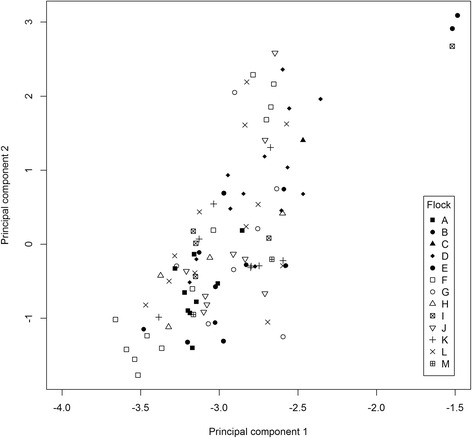
Population structure of Gute sheep genotyped with seven microsatellites using principal component analysis. Principal components 1 and 2 for individuals were plotted (shapes indicate which flock they were from)

A total of 94 animals (19 males and 75 females) from 13 flocks were successfully genotyped (Additional file 1: Table S1). All of the 94 genotyped animals had horns (which is typical for this breed). Average tail length was 14.2 cm (standard deviation: 1.45) with tail length ranging from 11 cm to 18 cm. The average inbreeding coefficient estimated from the pedigree was 0.018. The number of alleles per microsatellite marker ranged from three to seven. Five of seven of the microsatellite markers were not in Hardy Weinberg Equilibrium due to excess heterozygosity (Additional file 1: Table S2). On average, flocks had excess heterozygosity (average F_IS_ = -0.1102) and high diversity within and among flocks (1-Q_INTRA_ = 0.7102 and 1-Q_INTER_ = 0.6437) (Additional file 1: Table S3). This was also verified by the analysis of individual animals, which showed excess heterozygosity and high diversity within and among individuals (F_IS_ = -0.0561, 1-Q_INTRA_ = 0.7190 and 1-Q_INTER_ = 0.6808). Average Ritland inbreeding calculated for the 94 animals was low (F = 0.018). Multilocus heterozygosity and inbreeding calculated from microsatellites was highly correlated (0.90). The heterozygosity calculated using both methods was, on average, higher than expected (1.01845 and -0.03931). Inbreeding calculated from the pedigree had low correlations with both multilocus heterozygosity and inbreeding calculated from microsatellite markers (0.36 and 0.29). The average harmonic mean effective population size estimated from microsatellites has decreased by 4.2 (92.5 to 88.3) in the past five generations (~15 years). Using principal component analysis (PCA) to detect population structure revealed that individuals from all thirteen flocks generally cluster together in one group (Fig. 3). There were three individuals who were separate from the main cluster however these individuals were missing the same microsatellite genotypes and their position in the plot in Fig. 3 is likely a result of this.

## Discussion

We present the first analyses of genetic diversity of the Gute sheep population in Sweden. Pedigree and microsatellite genotype estimations of inbreeding were consistent with a breeding program aiming at reducing inbreeding.

Previous studies have suggested that Gute sheep is a unique breed with low heterogeneity [3, 4], which we could not verify in our study. Tapio et al. estimated inbreeding in 32 north European sheep breeds, including Gute sheep, using microsatellite markers [3]. All breeds in their study had greater F_IS_ estimates than our estimate for Swedish Gute sheep and many of the F_IS_ estimates indicated inbred populations (including for their Gute sheep population) [3]. The heterogeneity in our study was higher than expected in the population, both seen in the multilocus heterozygosity (1.01845) and the fact that five out of seven microsatellite markers were not in Hardy Weinberg equilibrium because of excess heterozygosity. In addition, inbreeding calculated from the entire pedigree, from the pedigree of the 94 individuals and from their microsatellite markers was low. The following could have contributed to differences in results: our diversity and inbreeding estimates are from animals born after animals studied by Tapio et al. [3]. Furthermore, we used pedigree data from the entire registered Swedish population and genotyped 94 animals from 13 different flocks with microsatellite genotypes while only 20 animals had been genotyped in the previous study [3]. Finally, we only used seven microsatellite markers compared to the 25 used by Tapio et al. [3] and this is a weakness of our study.

Inbreeding estimated based on data from the entire pedigree was more than twice the estimated inbreeding from the sample population (0.038 versus 0.018) which could be due to the amount of pedigree information available for each animal. The entire pedigree is more complete and has more depth: the average depth of the entire population pedigree in generations was 6.06 compared in contrast with 5.41 for the sample population. The founders of a pedigree are assumed to be unrelated and when information from more generations is available, there could be more relationships accounted for. Nevertheless, both estimates of inbreeding were low and our hypothesis is, that this could be because neither of the datasets could account for bottlenecks occurring earlier in time (ie. Before pedigree information was collected). However, the estimation of inbreeding from microsatellite genotypes of the sample population, should be able to account for historical inbreeding, but the estimation of inbreeding from microsatellite data was also low (Ritland inbreeding = -0.03931). We are therefore confident, that our estimation of a low inbreeding reflects reality.

This was also verified by the further analysis, which indeed indicated the existence of bottlenecks in the population. The difference between the effective number of founders and the effective number of ancestors indicated that bottlenecks have occurred in this population and the difference of half the effective population size and the effective number of founder genomes indicated that drift has been accelerating since the founder population.

Previous studies found heterozygote deficiency in European sheep breeds (including North European short-tailed breeds), which was thought to be due to subdivision among flocks creating the Wahlund effect and due to non-random mating [3, 18–21]. In contrast we found that there was excess heterozygosity in Swedish Gute sheep. Excess heterozygosity in microsatellites in a population can be due to the structure of the breeding program, where unrelated animals are bred together as typically found in a population within a conservation program. For the sample population genotyped, 95% of animals had parents with different flock ID numbers indicating that they were born in different flocks, consistent with the breeding of animals that are unrelated. The use of pedigree information in a conservation breeding program has been shown to be a very powerful tool for maintaining genetic diversity and low inbreeding [22] and our study showed similar results.

Heterozygosity and inbreeding calculated from microsatellite markers were highly correlated with each other but were not with inbreeding calculated from the pedigree. Regardless, we can conclude from these results that there was low inbreeding. Multilocus heterozygosity is not a good indicator of inbreeding calculated from a pedigree because it is not highly correlated unless there is a large amount of variation in inbreeding in a population [23] and heterozygosity can be lost at a different rate than inbreeding is gained in a population [24]. Additionally, correlation between these estimates can be lower because of amount and quality of information in the recorded pedigree. In a study of Finnsheep, estimates of inbreeding were correlated, however, estimates were not as correlated in one subpopulation of Finnsheep (grey individuals) and the authors believed this was in part because of their less complete pedigree [25].

The effective population size estimated from microsatellites was 88.3, which was lower than the effective population size calculated from the whole pedigree (155.4). However both of these estimates fall well within or even above the recommended effective population size for small populations, which should be at least between 50 and 100 [26, 27]. On the other hand it is still important to maintain the conservation breeding program and monitor progress because in order to maintain genetic variation in the long term, an effective population size of at least 500 is needed [28]. Nevertheless, the comparison of our results with that of earlier studies suggests that the current conservation strategy in the Swedish Gute sheep population is successful and should be maintained.

## Conclusions

The Swedish Gute sheep population can be an example of conservation for other small local breeds.

Despite evidence of historical bottlenecks, inbreeding estimates from the pedigree of all registered Gute sheep in Sweden and from the pedigree and microsatellite genotypes of a sample of the population indicated that the level of inbreeding was low and that inbreeding did not increase over the last six years of data analysed. While the Swedish Gute sheep population has an effective population size that is good for small populations, the current conservation program and monitoring of the average inbreeding should continue.

## Additional files

Additional file 1: Contains the following three tables. **Table S1.** 94 Gute sheep microsatellite genotyped by flock and sex. **Table S2.** Microsatellite markers, number of alleles and Hardy Weinberg equilibrium. **Table S3.** Gene diversity and inbreeding by flock over all loci with at least two individuals typed. (DOCX 21 kb)
Additional file 2: Contains the microsatellite genotypes for the 94 gute sheep. (TXT 22 kb)
Additional file 3: Contains the year of birth and horn and tail phenotypes for the 94 gute sheep with microsatellite genotypes. (TXT 2 kb)
