# Supplementary material for: Estimation of genetic diversity in Gute sheep: pedigree and microsatellite analyses of an ancient Swedish breed
Source: Hereditas. 2017 Jan 30;154:4. doi: 10.1186/s41065-017-0026-4 (PMC5282709; doi:10.1186/s41065-017-0026-4)
Supplement: Additional file 1: — Contains the following three tables. Table S1. 94 Gute sheep microsatellite genotyped by flock and sex. Table S2. Microsatellite markers, number of alleles and Hardy Weinberg equilibrium. Table S3. Gene diversity and inbreeding by flock over all loci with at least two individuals typed. (DOCX 21 kb) [file 41065_2017_26_MOESM1_ESM.docx]

**Estimation of genetic diversity in Gute sheep: pedigree and microsatellite analyses of an ancient Swedish breed**

**Table S1:** 94 Gute sheep microsatellite genotyped by flock and sex

| Flock | N | N males | N females |
| --- | --- | --- | --- |
| A | 9 | 0 | 9 |
| B | 6 | 0 | 6 |
| C | 1 | 0 | 1 |
| D | 13 | 3 | 10 |
| E | 6 | 6 | 0 |
| F | 12 | 9 | 3 |
| G | 7 | 0 | 7 |
| H | 4 | 0 | 4 |
| I | 5 | 0 | 5 |
| J | 9 | 1 | 8 |
| K | 8 | 0 | 8 |
| L | 12 | 0 | 12 |
| M | 2 | 0 | 2 |
| Total (13 flocks) | 94 | 19 | 75 |

**Table S2:** Microsatellite markers, number of alleles and Hardy Weinberg equilibrium

| Microsatellite marker | Number of alleles | Hardy Weinberg test p value (standard error)* |
| --- | --- | --- |
| INRA005 | 7 | 0.2462 (0.0064) |
| INRA023 | 5 | 0.0436 (0.0017) |
| INRA063 | 6 | 0.0214 (0.0016) |
| INRA172 | 6 | 0.0026 (0.0003) |
| MAF214 | 3 | 0.4141 (0.0041) |
| MAF65 | 4 | 0.0050 (0.0004) |
| McM527 | 4 | 0.0005 (0.0002) |
| Total |  | 0.0000 (0.0000) |

*p<0.05: heterozygote excess

**Table S3:** Gene diversity and inbreeding by flock over all loci with at least two individuals typed

| Flock | Gene diversity within individuals (1-Q_INTRA_) | Gene diversity among individuals (1-Q_INTER_) | Inbreeding of individuals compared to population (F_IS_) |
| --- | --- | --- | --- |
| A | 0.7778 | 0.6379 | -0.2193 |
| B | 0.7381 | 0.7000 | -0.0544 |
| C | - | - | - |
| D | 0.7471 | 0.6379 | -0.1712 |
| E | 0.7353 | 0.6696 | -0.0981 |
| F | 0.6000 | 0.5839 | -0.0275 |
| G | 0.6042 | 0.6344 | 0.0476 |
| H | 0.5714 | 0.5298 | -0.0787 |
| I | 0.8710 | 0.6519 | -0.3361 |
| J | 0.8197 | 0.6230 | -0.3158 |
| K | 0.7455 | 0.6245 | -0.1938 |
| L | 0.7407 | 0.6455 | -0.1476 |
| M | 0.5714 | 0.7857 | 0.2727 |
| Average | 0.7102 | 0.6437 | -0.1102 |
